# Supplementary material for: Empirical methods for controlling false positives and estimating confidence in ChIP-Seq peaks
Source: BMC Bioinformatics. 2008 Dec 5;9:523. doi: 10.1186/1471-2105-9-523 (PMC2628906; doi:10.1186/1471-2105-9-523)
Supplement: Additional File 1 — A variety of html documents from the USeq web site detailing the available applications, their best usage, output file type descriptions, command line menus, etc. [file 1471-2105-9-523-S1.zip › USeqUserGuides/JavaDoc.zip!/JavaDoc/gata/aligner/LocalAlignment.html]

LocalAlignment


|  |  |  |
| --- | --- | --- |
| **Overview** \| **Package** \| **Class** \| **Use** \| **Tree** \| **Deprecated** \| **Index** \| **Help** | |  |
| **PREV CLASS**   **NEXT CLASS** | **FRAMES**    **NO FRAMES**     **All Classes** |  |
| SUMMARY: NESTED \| FIELD \| CONSTR \| METHOD | DETAIL: FIELD \| CONSTR \| METHOD |  |


---


## gata.aligner Class LocalAlignment

```
java.lang.Object
  gata.aligner.LocalAlignment
```

**All Implemented Interfaces:**: java.io.Serializable

---

``` public class LocalAlignment extends java.lang.Object implements java.io.Serializable ```

**Author:**
:   nix
    Similar to but not exactly like Alignment obj, represents the Local Alignment returned by
    Blast. It's saved and made availible to GATAPlotter.

**See Also:**: Serialized Form

---

**Constructor Summary**

`LocalAlignment(java.lang.String seq_1,
int startSeq_1,
int stopSeq_1,
java.lang.String seq_2,
int startSeq_2,
int stopSeq_2,
int ori_,
AlignParams ap_,
int score_)`


| **Method Summary** | |
| --- | --- |
| `java.util.ArrayList` | `clearDups(java.util.ArrayList aligns)` |
| `AlignParams` | `getAP()` |
| `int` | `getLAScore()` |
| `java.lang.String` | `getLocalAlignString(int subStart, int subLength)` |
| `int` | `getOri()` |
| `java.util.ArrayList` | `makeAlignmentObjects(java.util.ArrayList alignNums)` |
| `int[][]` | `makeWindowsFromAlign()` |
| `java.util.ArrayList` | `mergeSubAligns(java.util.ArrayList winds)` |
| `java.util.ArrayList` | `optimizeScores(int[][] aligns)` |
| `void` | `printLocalAlignment()` |
| `java.util.ArrayList` | `processLocalAlignment()` |

**Methods inherited from class java.lang.Object**

`equals, getClass, hashCode, notify, notifyAll, toString, wait, wait, wait`

**Constructor Detail**

### LocalAlignment

```
public LocalAlignment(java.lang.String seq_1,
                      int startSeq_1,
                      int stopSeq_1,
                      java.lang.String seq_2,
                      int startSeq_2,
                      int stopSeq_2,
                      int ori_,
                      AlignParams ap_,
                      int score_)
```


**Method Detail**

### getAP

```
public AlignParams getAP()
```

---


### getOri

```
public int getOri()
```

---


### getLAScore

```
public int getLAScore()
```

---


### printLocalAlignment

```
public void printLocalAlignment()
```

---


### getLocalAlignString

```
public java.lang.String getLocalAlignString(int subStart,
                                            int subLength)
```

---


### processLocalAlignment

```
public java.util.ArrayList processLocalAlignment()
```

---


### makeWindowsFromAlign

```
public int[][] makeWindowsFromAlign()
```

---


### optimizeScores

```
public java.util.ArrayList optimizeScores(int[][] aligns)
```

---


### mergeSubAligns

```
public java.util.ArrayList mergeSubAligns(java.util.ArrayList winds)
```

---


### clearDups

```
public java.util.ArrayList clearDups(java.util.ArrayList aligns)
```

---


### makeAlignmentObjects

```
public java.util.ArrayList makeAlignmentObjects(java.util.ArrayList alignNums)
```


---


|  |  |  |
| --- | --- | --- |
| **Overview** \| **Package** \| **Class** \| **Use** \| **Tree** \| **Deprecated** \| **Index** \| **Help** | |  |
| **PREV CLASS**   **NEXT CLASS** | **FRAMES**    **NO FRAMES**     **All Classes** |  |
| SUMMARY: NESTED \| FIELD \| CONSTR \| METHOD | DETAIL: FIELD \| CONSTR \| METHOD |  |


---
